# HIF-1-dependent lipin1 induction prevents excessive lipid accumulation in choline-deficient diet-induced fatty liver

**DOI:** 10.1038/s41598-018-32586-w

**Published:** 2018-09-21

**Authors:** Takatomo Arai, Masako Tanaka, Nobuhito Goda

**Affiliations:** 1 Department of Life Sciences and Medical BioScience, Waseda University School of Advanced Science and Engineering, Tokyo, 162-8480 Japan; 2 Present Address: Takatomo Arai, Astellas Pharma Inc., Ibaraki, 305-8585 Japan

## Abstract

Adaptive responses to hypoxia regulate hepatic lipid metabolism, but their consequences in nonalcoholic fatty liver disease (NAFLD) are largely unknown. Here, we show that hypoxia inducible factor-1 (HIF-1), a key determinant of hypoxic adaptations, prevents excessive hepatic lipid accumulation in the progression of NAFLD. When exposed to a choline-deficient diet (CDD) for 4 weeks, the loss of hepatic *Hif-1α* gene accelerated liver steatosis with enhanced triglyceride accumulation in the liver compared to wild-type (WT) livers. Expression of genes involved in peroxisomal fatty acid oxidation was suppressed significantly in CDD-treated WT livers, whereas this reduction was further enhanced in *Hif-1α*-deficient livers. A lack of induction and nuclear accumulation of lipin1, a key regulator of the PPARα/PGC-1α pathway, could be attributed to impaired peroxisomal β-oxidation in *Hif-1α*-deficient livers. The lipin1-mediated binding of PPARα to the acyl CoA oxidase promoter was markedly reduced in *Hif-1α*-deficient mice exposed to a CDD. Moreover, forced *Lipin1* expression restored the aberrant lipid accumulation caused by *Hif-1α* deletion in cells incubated in a choline-deficient medium. These results strongly suggest that HIF-1 plays a crucial role in the regulation of peroxisomal lipid metabolism by activating the expression and nuclear accumulation of lipin1 in NAFLD.

## Introduction

Nonalcoholic fatty liver disease (NAFLD) is a common chronic liver disease that is characterized by simple steatosis, steatohepatitis, hepatic fibrosis, and cirrhosis. NAFLD is associated with systemic metabolic disorders, including obesity, type II diabetes mellitus, atherosclerosis, and dyslipidemia, and is considered to be the hepatic component of metabolic syndrome. Accumulating evidence has suggested that insulin resistance, oxidative stress, and dysregulated adipocytokine production play critical roles in the development and progression of NAFLD^1^. However, no effective therapies have yet been established for the disease because of an incomplete understanding of its pathogenesis.

Chronic liver hypoxia has been implicated as a cause and/or consequence of NAFLD, and has also been associated with adverse disease prognosis. Previous reports from our and other laboratories revealed disease-associated hypoxia in murine livers that had been chronically exposed to high-fat diets^2,3^. This was associated with mitochondrial dysfunction, including impaired fatty acid oxidation, reduced electron transport chain activity, and increased reactive oxygen species (ROS) production^2^. In addition, NAFLD-induced cytochrome P450 2E1 consumes a large amount of oxygen to oxidize polyunsaturated fatty acids^4^. This increases ROS formation, which disrupts hepatic oxygen homeostasis. These hypoxic alterations might in turn accelerate hepatic lipid accumulation and inflammatory cell infiltration, forming a vicious cycle that results in irreversible fibrotic remodeling in the liver. Intermittent hypoxia with a high-fat diet also enhances hepatic steatosis with concomitant liver inflammation and lipid peroxidation^5^, further supporting the aggravating effects of tissue hypoxia on NAFLD. However, the pathological significance of liver hypoxia in NAFLD has not been fully elucidated.

Mammalian cells have evolved to adapt to lowered oxygen conditions by activating a master transcriptional regulator of the hypoxic response, hypoxia inducible factor (HIF)^6,7^. HIF is composed of two distinct subunits: oxygen-sensitive HIFα (HIF-1α, HIF-2α, and HIF-3α) and constitutively expressed HIFβ/aryl hydrocarbon receptor nuclear translocator (ARNT). HIFα is degraded rapidly under normoxic conditions due to high prolyl hydroxylase activity, which allows the von Hippel-Lindau (VHL) tumor suppressor protein to bind to HIFα. During hypoxia, the escape of HIFα from VHL recognition results in the activation of HIF-mediated transcription. The constitutive activation of HIFα in the liver by loss of the *Vhl* gene evokes massive lipid accumulation in an HIF-2α-dependent manner^8^. In contrast, liver-specific *Arnt* knockout mice express increased amounts of several lipogenic genes but deposit fewer triglycerides (TG) in their livers compared with control mice^9^. Fatty infiltration in response to a high-fat diet occurs comparably irrespective of hepatic *Hif-1α* gene status, although several genes involved in hepatic fatty acid metabolisms are suppressed in the mutant mice^10^. These observations clearly suggest that HIF-1 plays an indispensable role in the regulation of hepatic lipid metabolism, although distinct sets of HIF-target genes might be induced or suppressed to disrupt liver lipid homeostasis in an isoform-specific and a context-dependent manner.

In the present study, we revealed that loss of *Hif-1α* gene suppresses peroxisomal fatty acid oxidation by inhibiting induction of the peroxisome proliferator-activated receptor (PPAR)α coactivator, lipin1, and thereby aggravating lipid accumulation in the liver after chronic exposure to a CDD. These results suggest that HIF-1 plays an endogenous protective role during the development of NAFLD.

## Results

### Loss of the hepatic *Hif-1α* gene aggravates CDD-induced liver steatosis in mice

**Figure 1:**
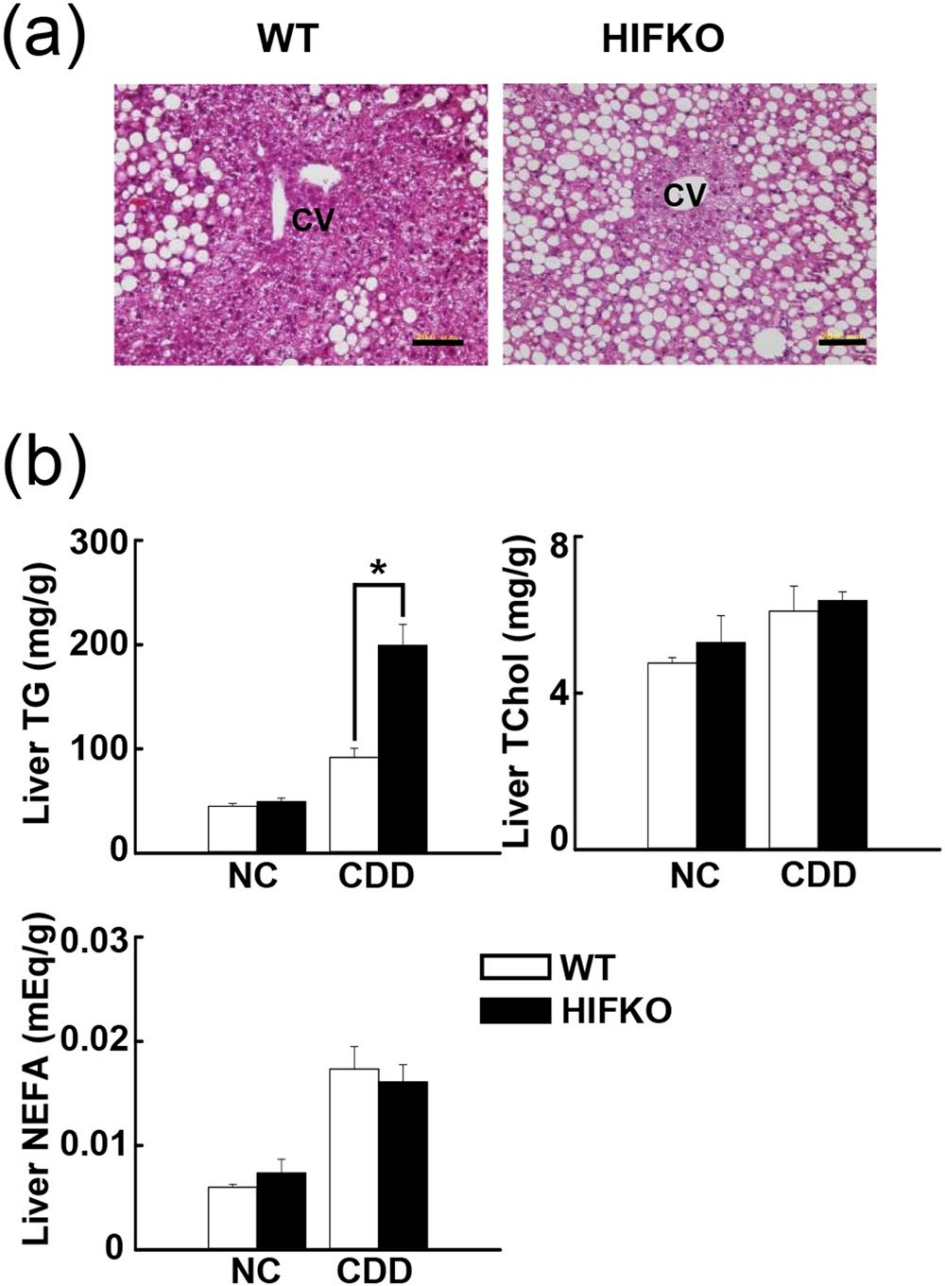
Hepatic *Hif-1α* deficiency enhances lipid accumulation in CDD-treated livers. (**a**) Representative H&E staining image of livers from WT and HIFKO mice fed a CDD. CV, central venule. Scale bar = 20 μm. (**b**) Liver lipid content in mice fed normal chow (NC) or a CDD. n = 5–10 mice per group. ^*^P < 0.05. TG, triglycerides; NEFA, non-esterified fatty acid.

We first investigated if exposure to a CDD for 4 weeks can activate HIF-1 transcriptional activity in mouse liver. Wild-type (WT) mice modestly increased HIF1α protein levels in liver by a CDD (Supplementary Fig. S1a). Liver expression of *Dmt1* (divalent metal transporter 1) and *Phd3* (prolyl hydroxylase domain-containing protein 3), well-known target genes of HIF-1, was elevated in WT mice exposed to a CDD, but these responses were almost abolished by inactivation of *Hif-1α* gene (Supplementary Fig. S1b). On the other hand, levels of *Vegf* (vascular endothelial growth factor), another target gene of HIF-1, were modestly, but significantly reduced in CDD-treated WT liver, while this was further enhanced in hepatocyte-specific *Hif-1α*-deficient (HIFKO) one (Supplementary Fig. S1b). We next examined effects of loss of hepatic *Hif-1α* gene on CDD-induced tissue damage, steatosis, and fibrosis in mouse liver. Serum levels of aspartate aminotransferase (AST) and alanine aminotransferase (ALT), indicative of tissue damage, were markedly elevated by a CDD, but were not different between WT and HIFKO mice (Supplementary Fig. S2a). Massive lipid accumulation occurred in the periportal hepatocytes of WT mice fed a CDD; these alterations were aggravated and expanded toward the midzonal region of lobules in HIFKO (Fig. 1a). Consistent with these findings, TG levels were elevated significantly in HIFKO livers compared with WT ones (Fig. 1b). The levels of liver non-esterified fatty acids (NEFA) increased substantially by CDD treatment in WT and HIFKO mice, but there was no significant difference between groups (Fig. 1b). Conversely, serum TG and NEFA levels did not show any remarkable change in response to a CDD, regardless of *Hif-1α* gene status (data not shown). Disruption of *Hif-1α* gene has been reported to reduce liver fibrosis when exposed to a high fat diets for 6 months^10^. However, in our model, sirius red staining of livers and gene expression analysis showed that liver fibrosis occurred comparably in both groups fed a CDD (Supplementary Fig. S2b,c). Collectively, these results suggest that deletion of hepatic *Hif-1α* gene selectively enhances fatty infiltration with increased liver TG content after exposure to a CDD.

### Decreased peroxisomal β-oxidation causes severe liver steatosis in HIFKO mice

**Figure 2:**
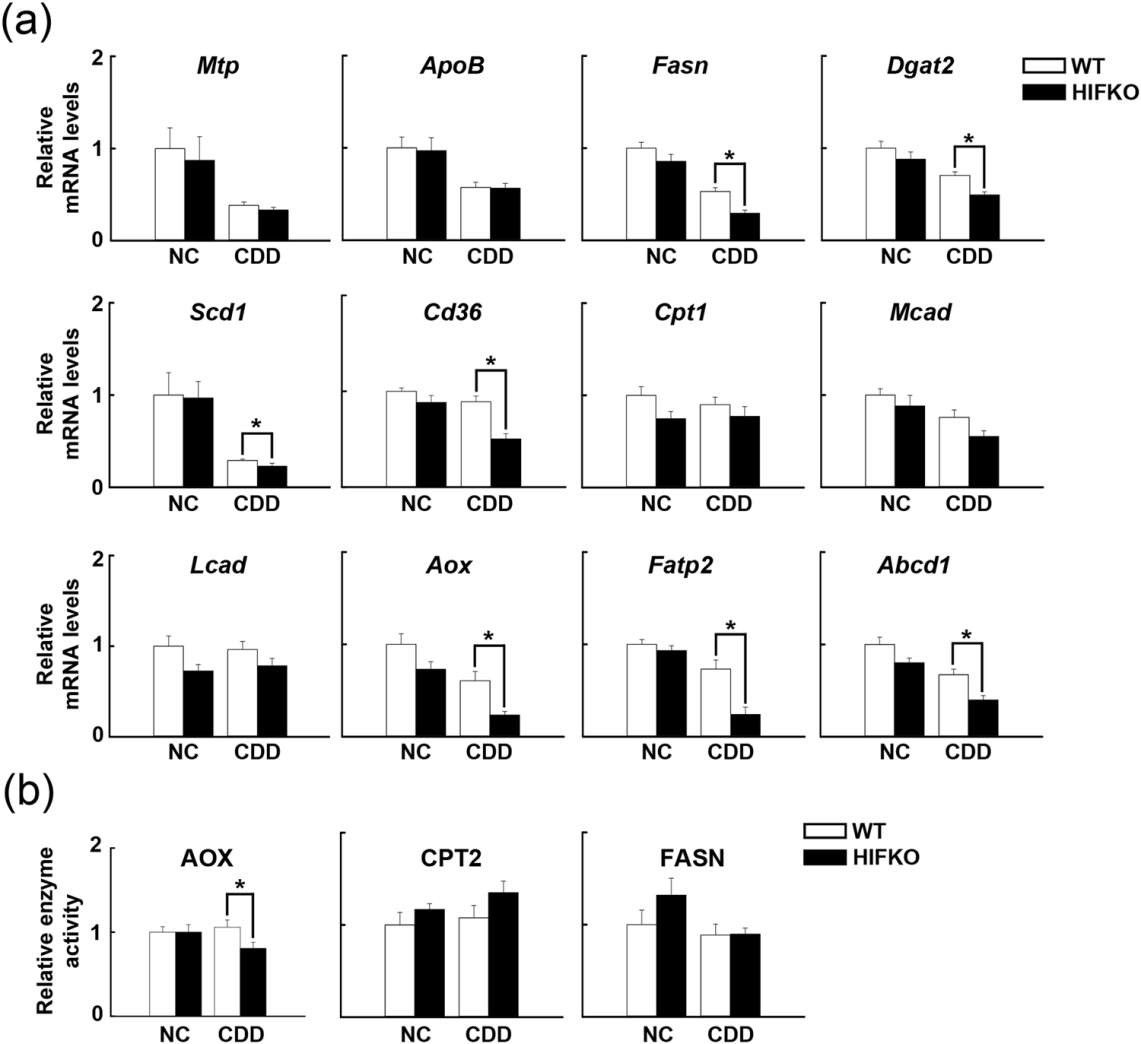
CDD-induced suppression of peroxisomal β-oxidation is aggravated in *Hif-1α*-deficient livers. (**a**) Analyses of the expression of hepatic lipid oxidation-related genes. NC, mice fed normal chow. n = 5–10 mice per group. *P < 0.05. (**b**) β-oxidation enzymatic activity in peroxisomes (AOX) and mitochondria (CPT2) in the livers of CDD-fed mice. NC, mice fed normal chow. n = 5–10 mice per group. *P < 0.05.

**Figure 3:**
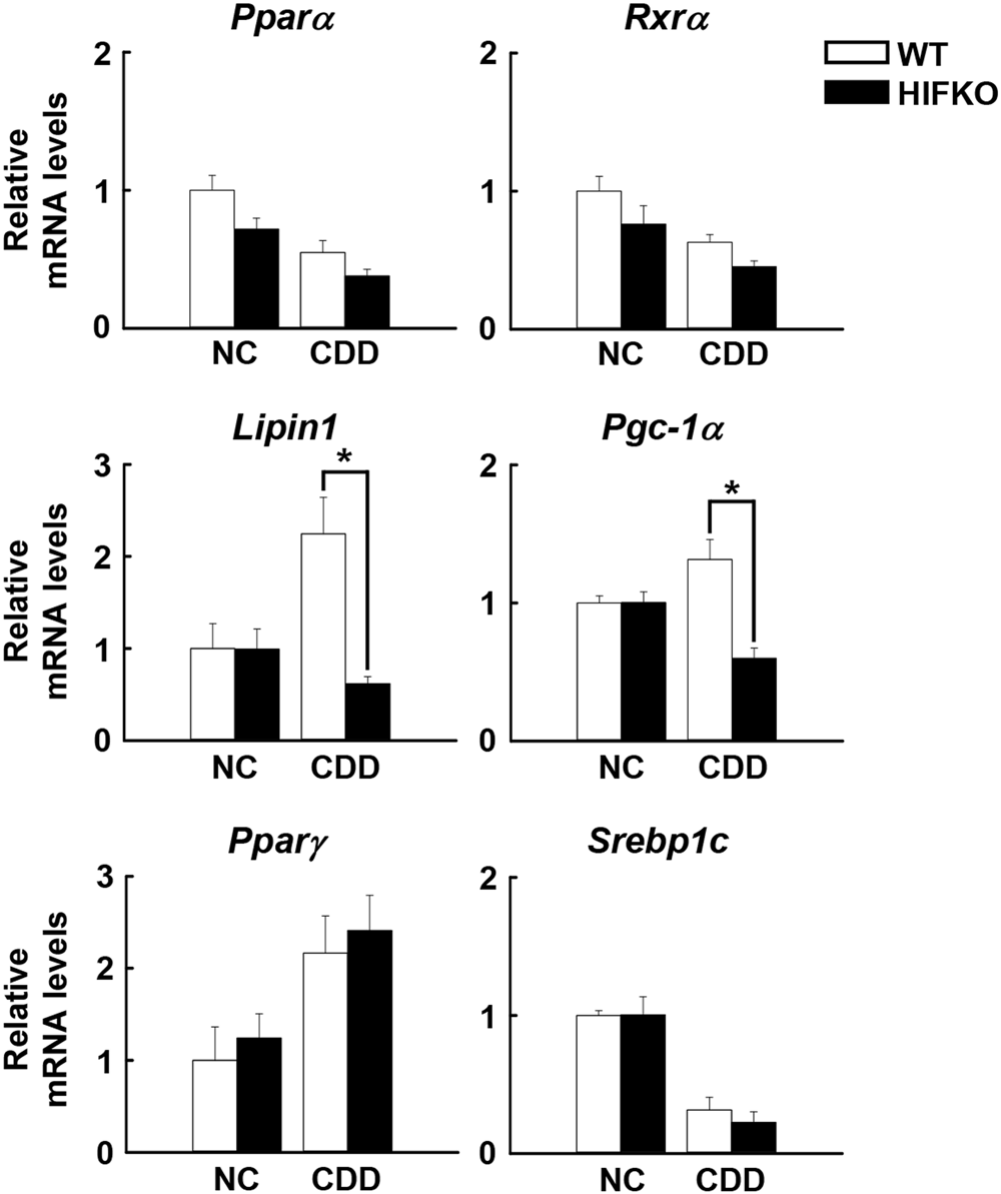
Loss of *Hif-1α* reduces the expression of PPARα/RXRα co-activators in CDD-treated livers. Quantification of mRNA of transcriptional factors and their associated transcriptional co-regulators for hepatic lipid metabolism in the livers of mice fed a CDD. NC, mice fed normal chow. n = 5–10 mice per group. ^*^P < 0.05.

We next examined the changes in lipid metabolism-related gene expression in CDD-induced fatty livers. Consistent with a previous report^11^, the expression of genes responsible for very low-density lipoprotein (VLDL) production, including *Mtp* (microsomal triglyceride transfer protein) and *ApoB* (apolipoprotein B), was reduced markedly by CDD treatment in WT and HIFKO mice (Fig. 2a). However, no differences were observed between groups. The suppression of *de novo* fatty acid synthesis-related genes such as *Fasn* (fatty acid synthase), *Dgat2* (diacylglycerol acyltransferase2), and *Scd1* (stearoyl CoA desaturase1) also occurred in the livers of WT mice fed a CDD (Fig. 2a), presumably due to an increased flux of NEFA into the liver (Fig. 1b). Surprisingly, HIFKO mice had further decreased expression of these lipogenic genes in response to a CDD. Consistent with these findings, the expression of a master regulator of lipogenic genes, *Srebp1c* (sterol regulatory element-binding protein1c), was reduced dramatically in steatotic livers compared with normal livers, and *Hif-1α* deficiency showed a trend toward further decreased expression of *Srebp1c* (Fig. 3). *CD36*, a member of the class B scavenger receptor responsible for transport of long-chain fatty acids, was significantly reduced in *Hif-1α*-deficient liver exposed to a CDD (Fig. 2a).

The expression of genes involved in mitochondrial fatty acid oxidation such as *Cpt1* (carnitine palmitoyltransferase1), *Mcad* (medium-chain acyl CoA dehydrogenase) and *Lcad* (long-chain acyl CoA dehydrogenase) was not changed significantly after exposure to a CDD (Fig. 2a). The loss of *Hif-1α* showed marginal effects on the expression of these genes. In contrast, the expression of peroxisomal fatty acid oxidation-related genes such as *Aox* (acyl CoA oxidase), *Fatp2* (fatty acid transport protein 2), and *Abcd1* (ATP-binding cassette, sub-family D1) was reduced significantly in CDD-treated WT livers, whereas these alterations were enhanced markedly by the deletion of hepatic *Hif-1α* (Fig. 2a). Collectively, these findings strongly suggest that the aggravated lipid accumulation in HIFKO mice could be partly attributed to severely impaired fatty acid oxidation in peroxisomes.

To further examine the importance of peroxisomal fatty acid oxidation in the aberrant lipid accumulation in HIFKO mice, *in vitro* enzymatic activity assays were performed. The hepatic activity of CPT2, which plays a role in the commitment step of mitochondrial β-oxidation, and *de novo* lipogenic activity, as assessed by FASN activity, were comparable between WT and HIFKO mice, even after exposure to a CDD (Fig. 2b). In contrast, deletion of *Hif-1α* caused a modest but significant reduction in AOX activity in CDD-treated livers (Fig. 2b), supporting the hypothesis that the enhanced suppression of peroxisomal β-oxidation by *Hif-1α* deficiency aggravates fatty infiltration in mice fed a CDD. However, expression of genes responsible for peroxisomal biogenesis (*Pex3*, *Pex11α* and *Pex14*) and functions (*Uox: urate oxidase*, and *catalase*) did not differ between WT and HIFKO mice even when exposed to a CDD (Supplementary Fig. S3a). Consistent with these results, protein levels of PEX14 and Catalase were comparable between the two groups (Supplementary Fig. S3b).

### The induction of *Lipin1*, a PPARα/PGC1α coactivator, is abolished in the aggravated fatty livers of HIFKO mice

PPARα is a transcription factor that regulates the expression of genes involved in peroxisomal fatty acid oxidation; its dysfunction causes severe hepatic steatosis^12^. We next assessed if the expression of *Pparα* and/or its transcriptional partner and coactivators is disturbed during the enhanced lipid accumulation in HIFKO mice fed a CDD. Choline deprivation significantly suppressed the levels of *Pparα* and *Rxrα* (retinoid X receptor α) in both WT and HIFKO mice (Fig. 3). The loss of *Hif-1α* diminished expression of a highly inducible transcriptional coactivator of PPARα, PPARγ coactivator-1α (*Pgc-1α*), markedly after exposure to a CDD (Fig. 3). However, the protein levels of PGC-1α were comparable between WT and HIFKO mice fed a CDD (data not shown). The expression of *Lipin1*, a key regulator of the PPARα/PGC-1α pathway, was induced significantly by choline deprivation in WT mice, whereas this induction was totally abolished in HIFKO mice (Fig. 3). There are two splice variants of *Lipin1*: *Lipin1α* and *Lipin1β*. The expression of hepatic *Lipin1β*, but not *Lipin1α*, increased in mice fed a CDD, which was dependent on HIF-1 expression (Supplementary Fig. S4). In addition, *Pparγ*, a key transcription factor for the pathogenesis of NAFLD, substantially increased by CDD treatment in both mice, but the levels were comparable between the groups (Fig. 3). Taken together, these results suggest that a lack of HIF-1 mediated *Lipin1* induction might stimulate aberrant lipid accumulation in the livers of mice fed a CDD by inhibiting PPARα transcriptional activity.

### Reduced lipin1 nuclear accumulation in the livers of HIFKO mice fed a CDD

**Figure 4:**
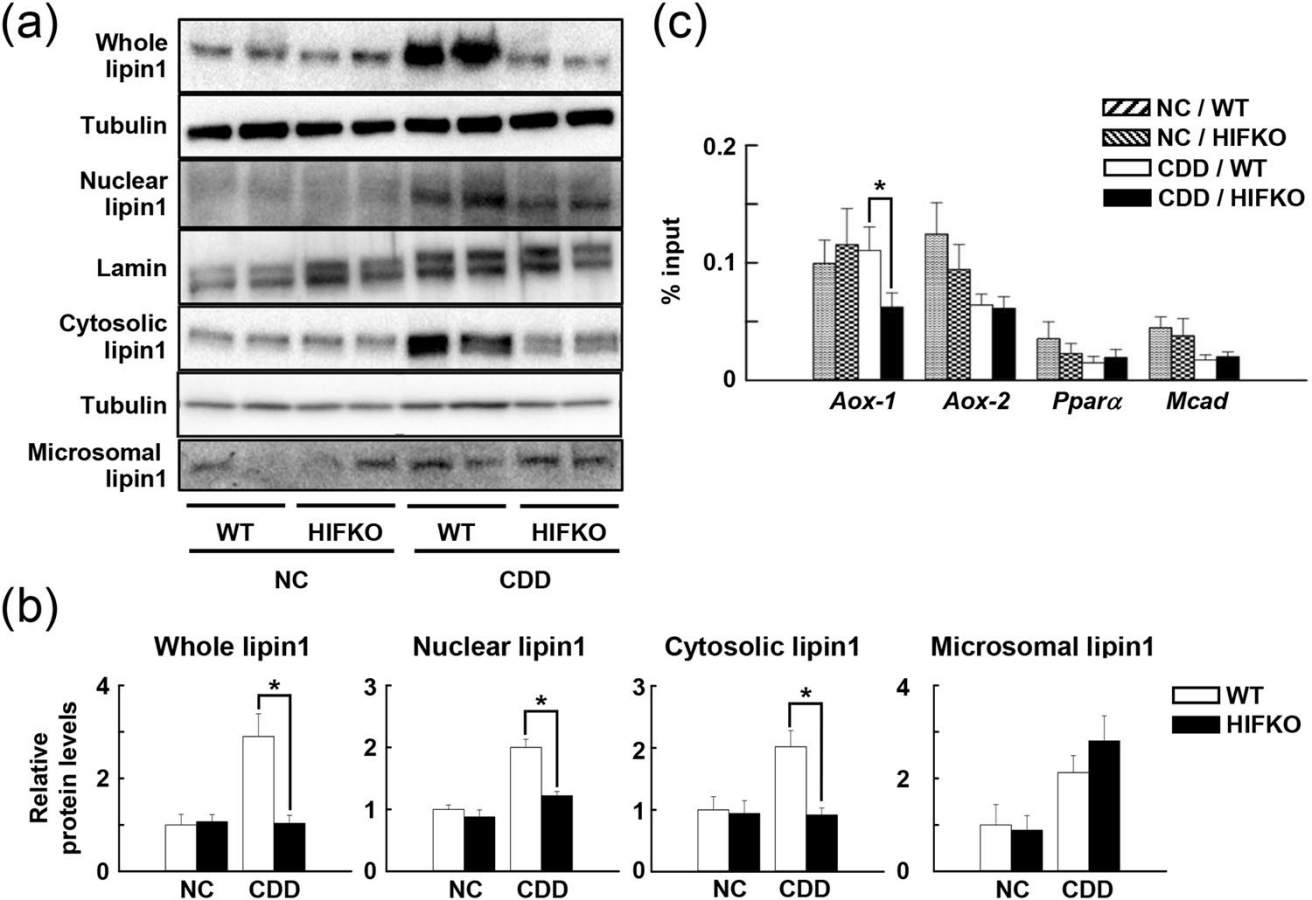
Deletion of *Hif-1α* attenuates the nuclear accumulation of Lipin1 in CDD-treated livers. (**a**) Representative immunoblots of lipin1 levels in different subcellular fractions of the livers of mice fed a CDD. NC, mice fed normal chow. (**b**) Protein levels of lipin1 in different subcellular fractions of the livers of mice fed a CDD quantified using densitometry; n = 5–7 mice per group. ^*^P < 0.05. (**c**) ChIP-qPCR analysis of lipin1 levels at the promoter of PPARα target genes in the livers of mice fed a CDD; n = 7–8 mice per group. ^*^P < 0.05.

Lipin1 exerts different functions in response to nutrients depending on its localization; endoplasmic reticulum (ER)-associated lipin1 acts as a lipid phosphatase that is essential for the synthesis of TG, whereas nuclear lipin1 functions as a coactivator for PPARα/PGC1α^13^. The total and cytosolic lipin1 protein levels increased significantly by a CDD in WT livers, whereas this induction was completely abolished in *Hif-1α*-deficient livers (Fig. 4a,b). ER-localized lipin1 levels were comparable between WT and HIFKO mice that had been fed a CDD (Fig. 4a,b), although microsomal lipin1 increased in response to a CDD in both groups. In contrast, nuclear lipin1 levels were elevated substantially in the livers of mice fed a CDD compared with normal livers, but the loss of *Hif-1α* abolished these alterations completely (Fig. 4a,b). These results suggest that HIF-1 induces lipin1 expression in response to a CDD, accumulating nuclear lipin1 and thereby sustaining peroxisomal fatty acid oxidation.

### Loss of *Hif-1α* abolishes the lipin1-mediated binding of PPARα to the *Aox* promoter region

We next investigated if the inhibition of increase in nuclear lipin1 accumulation resulted in decreased PPARα transcriptional activity in the livers of CDD-fed HIFKO mice using ChIP assays with anti-lipin1 antibodies. Two putative PPARα binding sites in *Aox* gene promoter, *Aox-1* and *Aox-2*, were found to be enriched constitutively in both WT and HIFKO mice fed normal chow (Fig. 4c). Feeding with a CDD showed a trend toward decreased enrichment of *Aox-2* in the liver irrespective of *Hif-1α* gene status. On the other hand, CDD treatment greatly reduced the binding of lipin1 to *Aox-1* in HIFKO, but not WT mice (Fig. 4c). In addition, CDD feeding and *Hif-1α* gene status did not affect the lipin1-mediated binding to the PPAR response element of *Pparα* and *Mcad* gene (Fig. 4c). Taken together, these results suggest that sustained PPARα-mediated transcription of peroxisomal fatty acid oxidation-related genes inhibits aberrant lipid accumulation in response to a CDD by activating lipin1 in a HIF-1-dependent manner.

### Forced *Lipin1β* expression inhibits *Hif-1α* deletion-induced aberrant lipid accumulation

**Figure 5:**
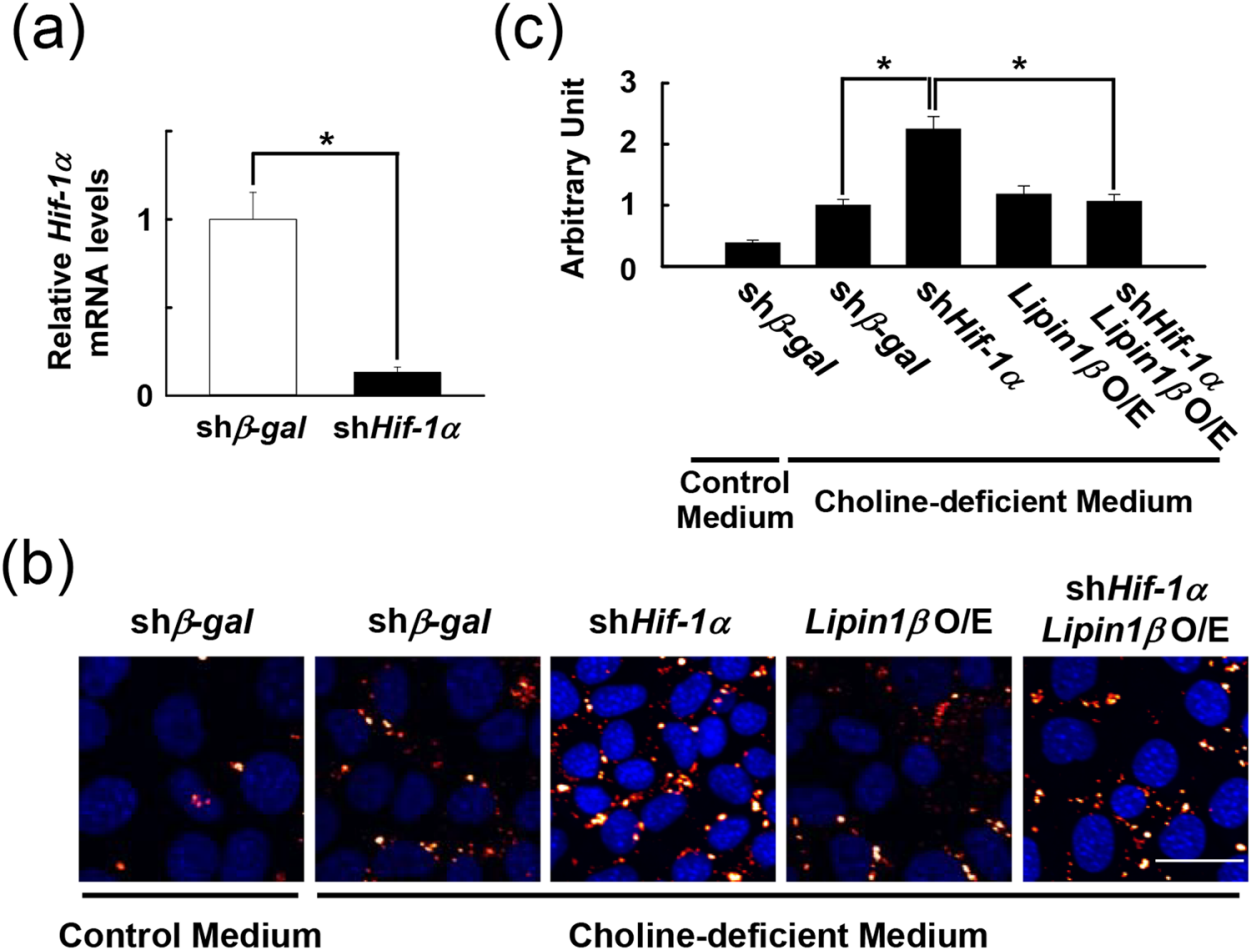
Forced *Lipin1* expression abolishes choline deprivation-induced lipid accumulation in cells lacking *Hif-1α*. (**a**) Quantification of *Hif-1α* mRNA by qPCR in AML12 cells infected with adenovirus expressing sh*Hif-1α*; n = 3 per group. ^*^P < 0.05 (**b**) Nile Red staining of AML12 cells exposed to choline-deficient medium. Lipid accumulation (red) is enhanced in cells lacking *Hif-1α* (middle), but reduced by *Lipin1β* overexpression (right). DAPI staining is shown in blue. Scale bar = 25 μm. (**c**) Densitometric quantification of lipid accumulation in A; n = 5–7 plates per group. ^*^P < 0.05.

Choline deficiency enhanced lipid deposition in cultured AML12 hepatocytes *in vitro* (Fig. 5b,c), confirming the cell-autonomous effects of choline deprivation on lipid accumulation. Lipin1 protein expression increased modestly, and was accumulated predominantly in the nuclei after exposure to choline-deficient medium (Supplementary Fig. S5a,c). In addition, we observed HIF-1α protein also increased modestly in cells by choline deprivation (Supplementary Fig. S5b). To confirm effects of loss of *Hif-1α* on choline deficiency-stimulated lipid accumulation, cells were infected with shRNA adenovirus against *Hif-1α*. Expression of *Hif-1α* significantly decreased by treating with an adenovirus expressing sh*Hif-1α* (Fig. 5a). Consistent with the *in vivo* results, inactivation of *Hif-1α* aggravated the lipid accumulation and decreased lipin1 expression in cells treated with choline-deficient medium compared with sh*β-galactosidase*-infected control cells (Fig. 5b,c, Supplementary Fig. S5a). This aberrant lipid deposition was abolished completely by forced expression of *Lipin1β* (Fig. 5b,c). In addition, suppression of *Lipin1* by introducing siRNA against *Lipin1* gene alone was observed to successfully enhance lipid accumulation in response to choline deficiency (Supplementary Fig. S5d,e). Collectively, these results support the importance of lipin1 expression in the HIF-1-mediated protection against lipid deposition.

## Discussion

Excessive lipid accumulation in hepatocytes is a hallmark of NAFLD. The metabolic alterations in NAFLD not only interfere with intracellular oxygen diffusion but also prevent the delivery of sufficient oxygen by narrowing the sinusoidal blood vessels in liver acinus^2,3^. The induction of Cyp2E1 expression in diet-induced steatosis has been well-documented in both rodents and humans^4^. This increased expression stimulates hepatic oxygen consumption and increases ROS production, which further impairs oxygen homeostasis in NAFLD livers. These metabolic and structural changes result in chronic liver hypoxia, and thereby induce varied adaptive responses to hypoxic stress by activating HIF-1 expression in the liver. However, the effect of this pathophysiological activation in NAFLD remains largely unclear. In the current study, we have demonstrated that the loss of *Hif-1α* in hepatocytes aggravates lipid infiltration in the liver in response to a CDD by reducing VLDL secretion and impairing peroxisomal β oxidation, the latter of which could explain the further increased lipid accumulation in the livers of HIFKO mice fed a CDD. These metabolic disturbances occur exclusively via the lack of HIF-1-mediated lipin1 expression and the resulting decrease in PPARα/PGC-1α-dependent expression of the genes involved in peroxisomal lipid metabolism.

The results of the current study clearly demonstrate that loss of *Hif-1α* in the liver augments hepatic steatosis in mice fed a CDD. This is consistent with our previous report showing that the alcoholic fatty liver is worsened in *Hif-1α*-deficient mice^14^. Although hepatic HIF-1 exerts anti-steatotic effects in these different disease conditions, the underlying mechanisms are distinct. In response to excessive alcohol exposure, HIF-1 suppresses *de novo* fatty acid synthesis and subsequent TG accumulation by inhibiting the aberrant activation of SREBP1c, a master lipogenic transcription factor. In contrast, HIF-1 prevents the massive reduction in fatty acid oxidation caused by choline deprivation by alleviating the diet-induced suppression of PPARα activity. This suggests that HIF-1 regulates hepatic lipid metabolism in a context-dependent manner. Given that lipid accumulation occurs in the pericentral and periportal regions of the liver acinus after exposure to alcohol-containing and choline-deficient diets, respectively, the different roles of HIF-1 in the regulation of hepatic lipid metabolism could be attributable to differences in its intrahepatic localization. Hepatocytes show distinct metabolic activity profiles depending on their localization in the liver acinus^15^: pericentral hepatocytes exhibit increased fatty acid synthesis, whereas periportal hepatocytes predominantly oxidize fatty acids. Therefore, it is possible that HIF-1 regulates lipid metabolism in an intrahepatic zone-specific manner by sensing and responding to distinct cellular microenvironments in different disease conditions.

Choline deficiency suppresses VLDL secretion and fatty acid oxidation, resulting in severe liver steatosis. A drastic reduction in the expression of *Mtp* and *ApoB*, which are essential for VLDL formation, occurs irrespective of *Hif-1α* gene status, suggesting that VLDL-related genes also play essential roles in the enhanced lipid accumulation in HIFKO livers. In contrast, the present findings revealed a marked reduction in *Aox*, *Fatp2*, and *Abcd1* expression as well as decreased AOX activity in HIFKO mice fed a CDD, providing evidence to suggest that aggravated fatty acid oxidation in peroxisomes leads to aberrant lipid accumulation in HIFKO mice. Long-chain and very long-chain fatty acids, which are preferred substrates for peroxisomal β-oxidation, have been implicated in the induction of steatohepatitis^16^. *Aox*-deficient mice develop severe steatosis spontaneously, in spite of the increased expression of mitochondrial β-oxidation-related genes^17^. This supports the current findings, whereby the aggravated CDD-induced inhibition of peroxisomal fatty acid oxidation caused aberrant lipid accumulation in *Hif-1α*-deficient livers. In contrast to a previous report showing impaired mitochondrial fatty acid oxidation in *Vhl*-deficient livers^8^, the ability of mitochondria to oxidize fatty acids was not affected severely in WT and HIFKO mice fed a CDD in the current study. This further highlights the importance of peroxisomal β-oxidation in the progression of CDD-induced fatty livers in HIFKO mice. The selective protection of HIF-1α against the CDD-induced suppression of peroxisomal fatty acid oxidation might help reduce the metabolic burden of excessive fatty acid oxidation in the mitochondria, resulting in decreased ROS production and the subsequent mitigation of ROS-induced disease progression toward steatohepatitis, as catalase enriched in peroxisome can efficiently reduce hydrogen peroxide produced in peroxisomal fatty acid oxidation.

Lipin1 is a multifunctional protein that plays roles in lipid metabolism^18^. Its phosphatidate phosphatase activity, which converts phosphatidate to diacylglycerol on the ER membrane, is a key process for *de novo* TG synthesis. In contrast, nuclear lipin1 serves as a transcriptional coactivator for PPARα and PGC-1α to induce expression of genes related to fatty acid oxidation. In the current study, exposing mice to a CDD resulted in the elevated microsomal lipin1 accumulation. This suggests a possible role for lipin1-mediated phosphatidate phosphatase activity in CDD-stimulated lipid accumulation in the liver. Consistent with a previous report showing hypoxia-induced HIF-1-dependent lipin1 expression^19^, CDD-induced lipin1 expression was abolished completely in HIFKO mice in the current study. However, HIFKO deteriorated excessive fatty infiltration without affecting microsomal lipin1 accumulation compared with CDD-treated WT mice, suggesting that lipin1-mediated TG synthesis is not critical for the aggravated phenotype in HIFKO mice. Instead, the loss of *Hif-1α* reduced nuclear lipin1 expression in the livers of CDD-fed mice compared with the WT liver, suggesting that the reduced nuclear accumulation of lipin1 could diminish its lipolytic transcriptional coactivator activity and thus augment lipid accumulation in HIFKO livers. A major isoform of *Lipin1* in the liver, *Lipin1β*, was reported to increase the expression of the PPARα target genes that are involved in fatty acid oxidation by associating directly with PGC-1α in the liver^13^. In addition, liver-specific *Lipin1*-deficient mice showed aggravated lipid accumulation by inhibiting VLDL excretion and fatty acid oxidation during the development of alcoholic liver steatosis^20^. Consistent with these reports, silencing of *Lipin1* gene alone enhanced choline deficiency-evoked lipid accumulation *in vitro*, further supporting the pathological importance of lipin1 induction against development of NAFLD.

Consistent with previous reports and the current *in vivo* results, knocking down *Hif-1α* in hepatocytes enhanced lipid accumulation and reduced lipin1 expression in choline-deficient medium. Moreover, the increased lipid deposition was abolished by the forced expression and enhanced nuclear accumulation of lipin1β, further highlighting the importance of nuclear lipin1 for the prevention of excessive and aberrant lipid accumulation in the liver. In fact, the increased nuclear lipin1 successfully sustained the binding of PPARα to a distal PPARα-responsive site in the *Aox* promoter of a CDD-treated WT liver, even though hepatic expression of *Pparα* and *Rxrα* substantially decreased. In addition, this hypothesis is supported by the current results showing the reduced association of lipin1 with the same PPARα-responsive site in the *Aox* promoter in the livers of HIFKO mice fed a CDD. However, this lipin1-mediated transcriptional regulation appeared to be selective for genes related to peroxisomal, but not mitochondrial, fatty acid oxidation because a putative PPARα-binding site in the *Mcad* promoter was not enriched by the CDD. This contradicts previous reports, where lipin1 activated several PPARα-target genes related to both peroxisomal and mitochondrial fatty acid oxidation in the liver^13^. Although the molecular mechanisms behind the differential binding of lipin1 to promoter regions remains unclear, the present findings suggest that the selective association between lipin1 and PPARα with the PPARα target genes that are involved in hepatic lipid metabolism after exposure to a CDD might play a role.

In conclusion, the current study revealed that HIF-1 protects against choline deprivation-induced NAFLD. Specifically, HIF-1-induced lipin1 expression inhibits aberrant lipid accumulation by preventing the suppression of peroxisomal fatty acid oxidation. Although these data suggest that HIF-1 might have therapeutic potential in liver steatosis during NAFLD, future studies are needed to clarify the pathological significance of HIF-1 in the development of non-alcoholic steatohepatitis and fibrosis because HIF-1 has diverse and complicated roles in the regulation of inflammation and tissue remodeling in different organs.

## Methods

### Animals and experimental procedures

Hepatocyte-specific *Hif-1α*-deficient (HIFKO) mice were generated by crossing HIF-1α^loxP/loxP^ (WT) mice with Alb-*Cre* transgenic mice, as described previously^21^. Male mice (5–6 weeks old) were exposed to a CDD (Oriental Yeast; Tokyo, Japan) for 4 weeks. All experiments were conducted in accordance with the Waseda University Animal Welfare Guidelines and approved by the Animal Experimentation Committee of the Waseda University (Protocol number: 2015-A032).

### Liver histology

Livers were fixed overnight in 10% phosphate-buffered formalin, and were embedded in paraffin. Five-micrometer-thick sections were then stained with hematoxylin and eosin (H&E) to assess lipid infiltration. Picro-sirius red staining was performed for collagen staining.

### Biochemical analyses

Hepatic lipid fractions were extracted using Folch solution (a mixture of 1 vol. of methanol and 2 vol. of chloroform), as described previously^14^. The amounts of TG, total cholesterol, and NEFA in liver and serum were then measured using colorimetric enzyme assays (Wako Pure Chemical Industries; Tokyo, Japan). Liver injury was assessed by measuring serum ALT and AST levels using a diagnostic kit (Wako Pure Chemical Industries).

### Quantitative polymerase chain reaction (qPCR)

Total RNA was extracted from the livers using an RNeasy Micro Kit (Qiagen; Tokyo, Japan). Complementary DNA was then synthesized using GoScript^TM^ Reverse transcription system (Promega; Tokyo, Japan) using 1 μg total RNA as the template. Quantitative PCR was performed using either GoTaq qPCR Master Mix (Promega) or FastStart TaqMan® Probe Master (Roche Diagnostics Japan; Tokyo, Japan) in a StepOnePlus^TM^ system (Applied Biosystems Japan; Tokyo, Japan). TaqMan gene expression assays (Applied Biosystems) and the sequences of the primers used in the current study are listed in Supplementary Table S1 in the supplemental material. Gene expression was normalized to either *β-actin* (for *Hif-1α*) or *18 S ribosomal RNA* (for other genes) as an internal control.

### Western blotting

Western blotting was performed using 50–100 μg of nuclear fraction, microsomal fraction, cytosolic fraction or whole extract of the liver, as described previously^14^. In some experiments, AML12 cells exposed to choline-deficient medium were lysed in 50 mM HEPES, pH 7.5, 150 mM NaCl, 1% CHAPS, 5 mM EDTA, and protease inhibitors (Nacalai Tesque, Kyoto, Japan), and resultant cell lysates (15 μg) were used. The expression of PGC-1α, lipin1, HIF-1α, lamin, and tubulin was detected using specific antibodies against the respective antigens as follows: PGC-1α (2178 S, Novus Biologicals; Littleton, CO, USA), lipin1 (AF3885, R&D Systems; Minneapolis, MN, USA), HIF-1α (36169 and 14179, Cell Signaling Technology, Danvers, MA, USA), lamin A/C (sc-20681, Santa Cruz Biotechnology; Santa Cruz, CA, USA), and tubulin (T9026, Sigma-Aldrich, St. Louis, MO, USA). Images of Western blots are shown in Supplementary Fig. S6.

### Enzyme assays

Livers (30 mg) were homogenized in 10 vol. of buffer (0.25 M sucrose, 1 mM EDTA, 3 mM Tris-HCl, pH7.2) supplemented with protease inhibitors (0.5 mM phenylmethylsulfonyl fluoride, 1 mM sodium orthovanadate, 1 μg/mL aprotinin, 1 μg/mL pepstatin A, and 2 μg/mL leupeptin). The lysates were then centrifuged at 500 × *g* for 10 min to obtain the supernatant as the peroxisome fraction. The peroxisome fraction was further centrifuged at 9000 × *g* for 10 min to collect the supernatant and pellet as the lipid synthesis fraction and the mitochondrial fraction, respectively. Hepatic AOX activity was determined based on the change in absorbance at 500 nm by incubating the peroxisome fraction (0.2 mg protein) in assay buffer (58 mM phosphate potassium buffer [pH 7.4], 10.6 mM phenol, 0.82 mM 4-aminoantipyrine, 10 μM FAD^+^, 4 units of peroxidase, 0.1 mM palmitoyl-CoA, and 0.2 mg albumin) for 5 min. To measure CPT2 and FASN activity in the liver, the mitochondrial (0.1 mg protein) and lipid synthesis fractions (0.15 mg protein) were mixed with the appropriate buffer (58 mM Tris-HCl [pH 8.0], 1.25 mM EDTA, 0.25 mM 5, 5′-dithiobis(2-nitrobenxoic acid), 0.04 mM palmitoyl-CoA, 0.1% TritonX-100, 1.25 mM l-carnitine for CPT2; and 0.1 M phosphate potassium buffer [pH 7.0], 0.2 mM EDTA, 0.3 mM NADPH, 0.05 mM acetyl-CoA, and 0.2 mM malonyl-CoA for FAS) for 3 min, and the light absorbance at 412 nm and 340 nm, respectively, was measured.

### Chromatin immunoprecipitation (ChIP)-qPCR

Livers (300 mg) were lysed in a buffer containing 10 mM Tris-HCl pH 7.5, 10 mM NaCl, 0.5% NP-40, and protease inhibitors. The samples were crosslinked using 1% formaldehyde for 10 min at room temperature, and were then prepared for ChIP as described previously^22^. The crosslinked samples were incubated with anti-lipin1 antibodies (R&D Systems) and protein G-Sepharose beads (Life Technologies Japan; Tokyo, Japan) overnight at 4 °C. The beads were then washed several times, and bound proteins were eluted in elution buffer (1% sodium dodecyl sulfate in 0.1 M NaHCO_3_). The elutes were treated with pronase (1 mg/mL; Roche) for 2 h at 42 °C, and were then were incubated at 65 °C overnight to remove the crosslinks. The samples were further purified using nucleospin extract II (TaKaRa; Tokyo, Japan). qPCR was performed using the primers listed in Table S1 in the supplemental material.

### Cell culture, adenovirus infection, and siRNA treatment

Normal mouse liver AML12 cells were purchased from ATCC (Manassas, VA, USA). The cells were cultured in Dulbecco’s modified Eagle medium (DMEM; Life Technologies) with or without choline chloride (Kohjin Bio, Saitama, Japan) containing penicillin (100 U/mL), streptomycin sulfate (100 μg/mL), and 10% fetal bovine serum for 72 h at 37 °C in 5% CO_2_. Cells were infected with adenovirus expressing either shRNA containing repeat mouse *Hif-1α* sequences (GCAACTGTCATATATAATACG), which were separated by a four-nucleotide spacer from the reverse complement of the same 21 nucleotide sequences (Life Technologies), or His-tagged-mouse *Lipin1* (Life Technologies) 24 h before experiments. Viruses encoding shRNA against *β-galactosidase* were used as a control. A multiplicity of infection of 100 for each virus was used for the virus infection. In some cases, cells were transfected with siRNA targeting for mouse *Lpin1* (50 nM, 5′-AGGUUGACGCCAAAGAAUAACCUGG-3′; Integrated DNA Technologies KK, Tokyo, Japan) using Lipofectamine 2000 (Life Technologies) 24 h before experiments.

### Nile Red staining and immunocytochemistry

After treatment with choline-deficient DMEM for 72 h, cells were fixed with 4% paraformaldehyde for 20 min at 4 °C. Lipid droplets were then visualized by staining cells with Nile Red (250 ng/mL) for 10 min followed by DAPI. Lipin1 expression was detected by incubating cells with anti-lipin1 antibodies followed by Alexa-Fluor® 488 goat anti-rabbit IgG (A-11034, Life Technologies). Cells were then observed using confocal microscopy (FV-1000, Olympus; Tokyo, Japan).

### Statistical analyses

Results are expressed as means ± SE. Statistical analyses were performed using Student’s t-test for all experiments. *P* values < 0.05 were considered to be significant.

## Electronic supplementary material

Supplementary Dataset1
