# Supplementary material for: HIF-1-dependent lipin1 induction prevents excessive lipid accumulation in choline-deficient diet-induced fatty liver
Source: Sci Rep. 2018 Sep 21;8:14230. doi: 10.1038/s41598-018-32586-w (PMC6155071; doi:10.1038/s41598-018-32586-w)
Supplement: Supplementary file 1 — Supplementary Dataset1 [file 41598_2018_32586_MOESM1_ESM.pdf]

Supplementary Information

HIF-1-dependent lipin1 induction prevents excessive lipid accumulation in choline-deficient diet-induced fatty liver

Takatomo Arai[#1], Masako Tanaka[1], Nobuhito Goda*[1]

[1]Department of Life Sciences and Medical BioScience, Waseda University School of Advanced Science and Engineering, Tokyo 162-8480, Japan

#Present address: Takatomo Arai, Astellas Pharma Inc., Ibaraki 305-8585, Japan

*Correspondence should be addressed to N.G. (goda@waseda.jp)

Table of Contents

Supplementary Figure S1……………………………………...….1

Supplementary Figure S2……………………………….……….…..2-3

Supplementary Figure S3……………………………………….....4

Supplementary Figure S4……………………………………….....5

Supplementary Figure S5……………………………………….....6-7

Supplementary Figure S6……………………………………….....8-11

Supplementary Table S1…………………………………………12

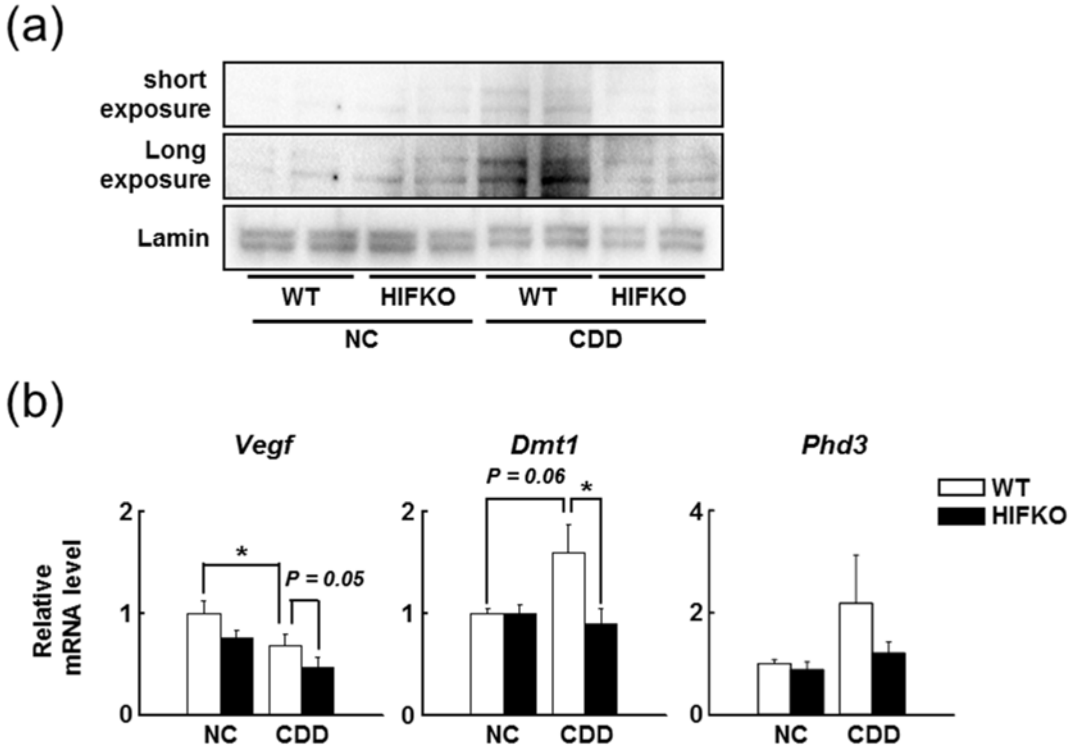

Supplementary Figure S1. Exposure to a CDD activates HIF-1α in mouse liver.

(a) Representative immunoblots of HIF-1α protein in livers of mice fed a CDD. NC, mice fed normal chow.

(b) Expression of well-known HIF-1 target genes in livers treated with a CDD. NC and CDD denote samples collected from mice fed normal chow and a CDD, respectively; n = 5-10 mice per group. *$P < 0.05$.

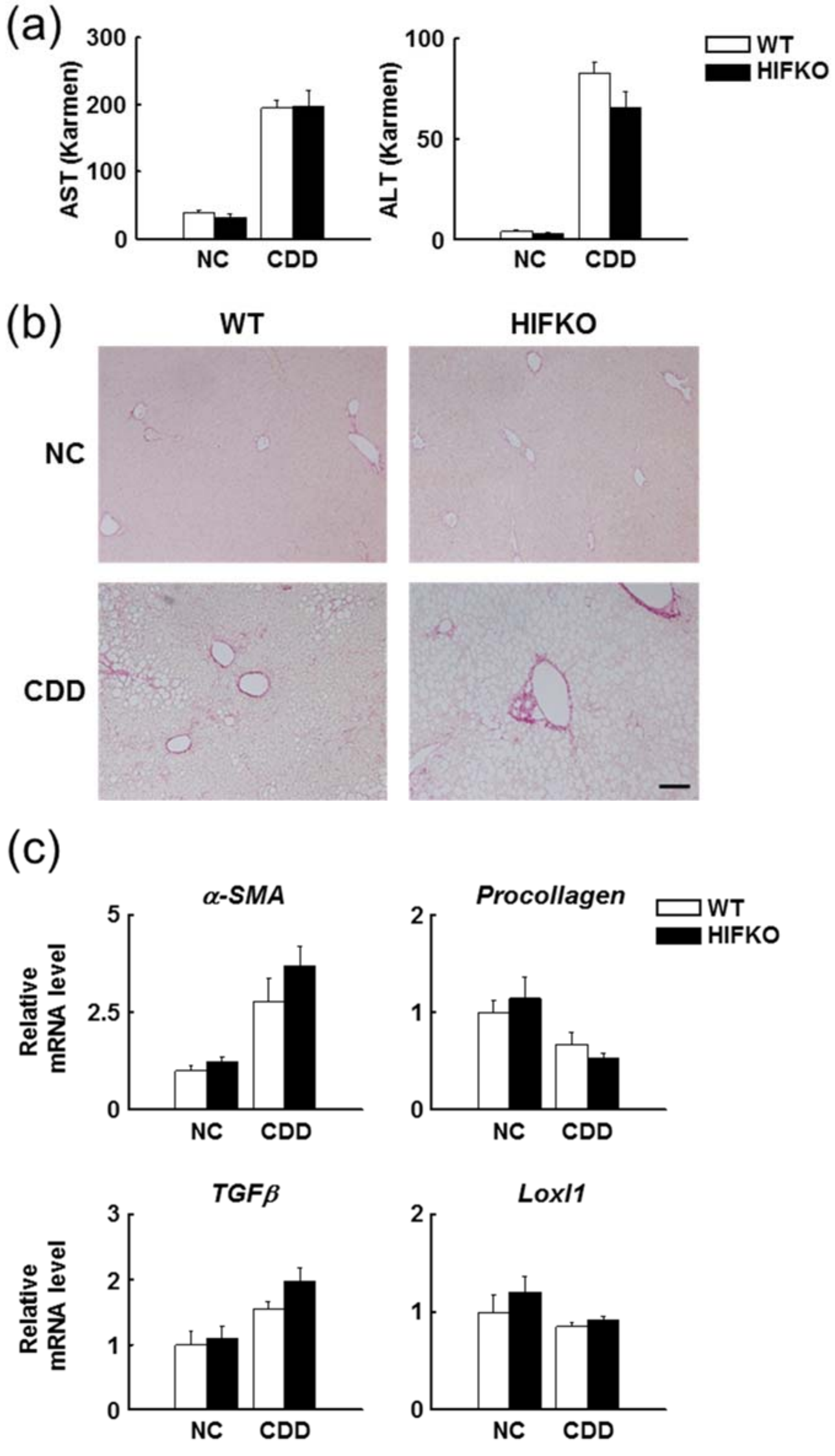

Supplementary Figure S2. Loss of *Hif-1α* gene shows little effects on liver fibrosis of mice fed a CDD.

(a) Serum levels of AST and ALT in mice fed a CDD. n = 5 mice per group.

(b) Representative sirius red staining image of livers of WT and HIFKO mice fed a CDD. Scale bar is 20 μm.

(c) Analysis of mRNA of liver fibrosis-related genes. NC and CDD denote samples collected from mice fed normal chow and a CDD, respectively; n = 5 mice per group.

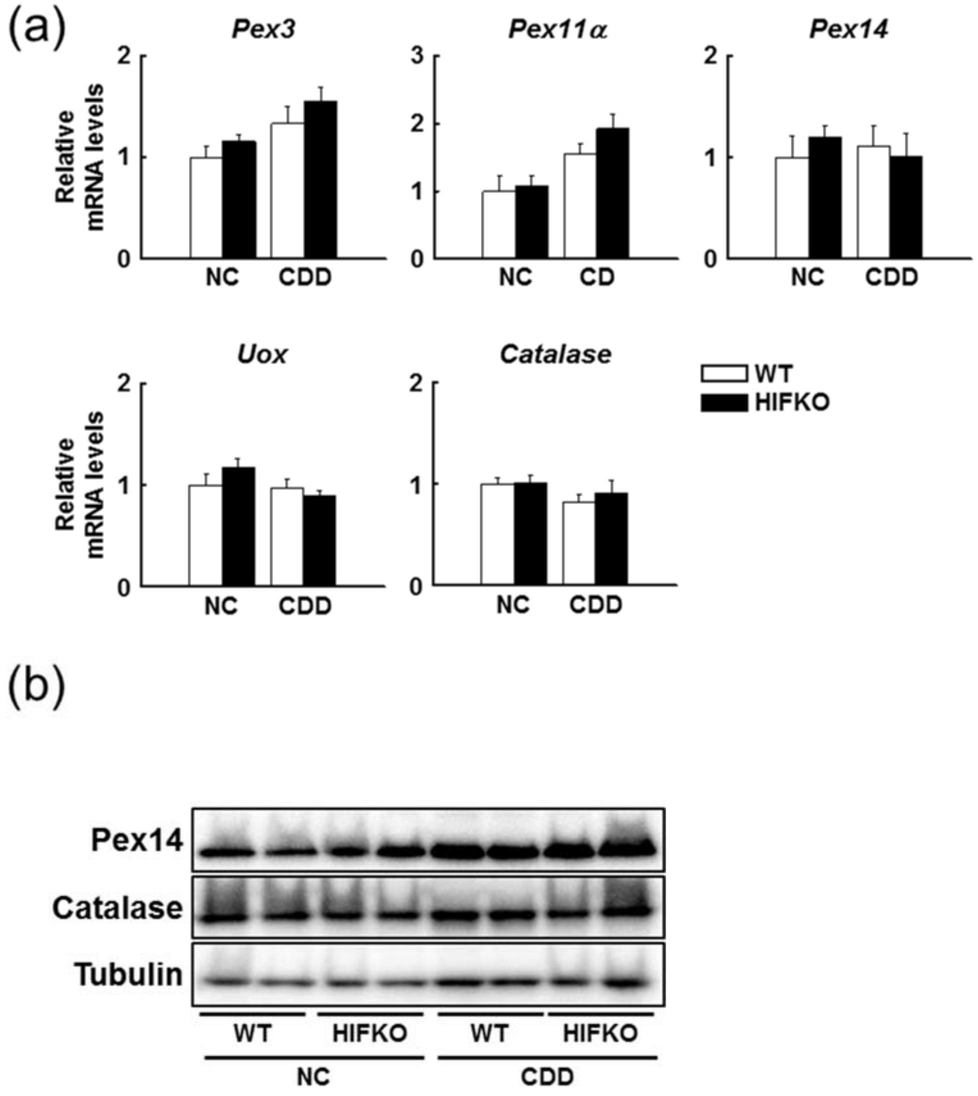

Supplementary Figure S3. Liver expression of peroxisome-related genes is comparable between WT and HIFKO mice fed a CDD.

(a) Analysis of mRNA of peroxisomal biogenesis and functions-related genes. NC and CDD denote samples collected from mice fed normal chow and a CDD, respectively; n = 5 mice per group

(b) Representative images of protein expression of Pex14 and Catalase in CDD-treated liver.

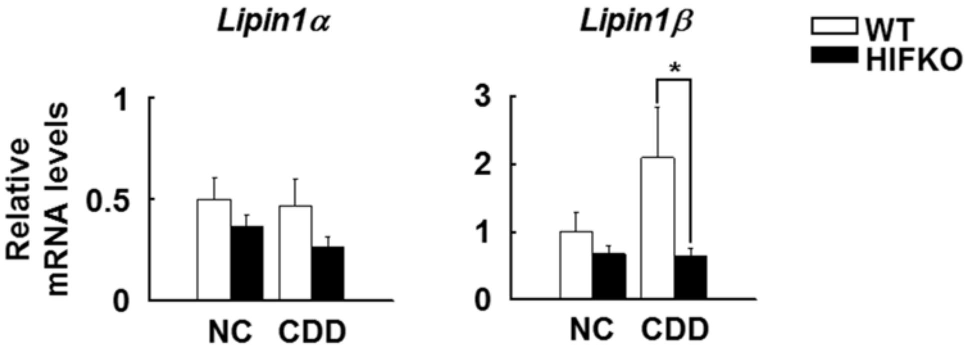

Supplementary Figure S4. *Lipin1β* is induced in a HIF-1-dependent manner in mice fed a CDD.

The mRNA was quantified. NC and CDD denote samples collected from mice fed normal chow and a CDD, respectively; n = 5 mice per group. *$P < 0.05$.

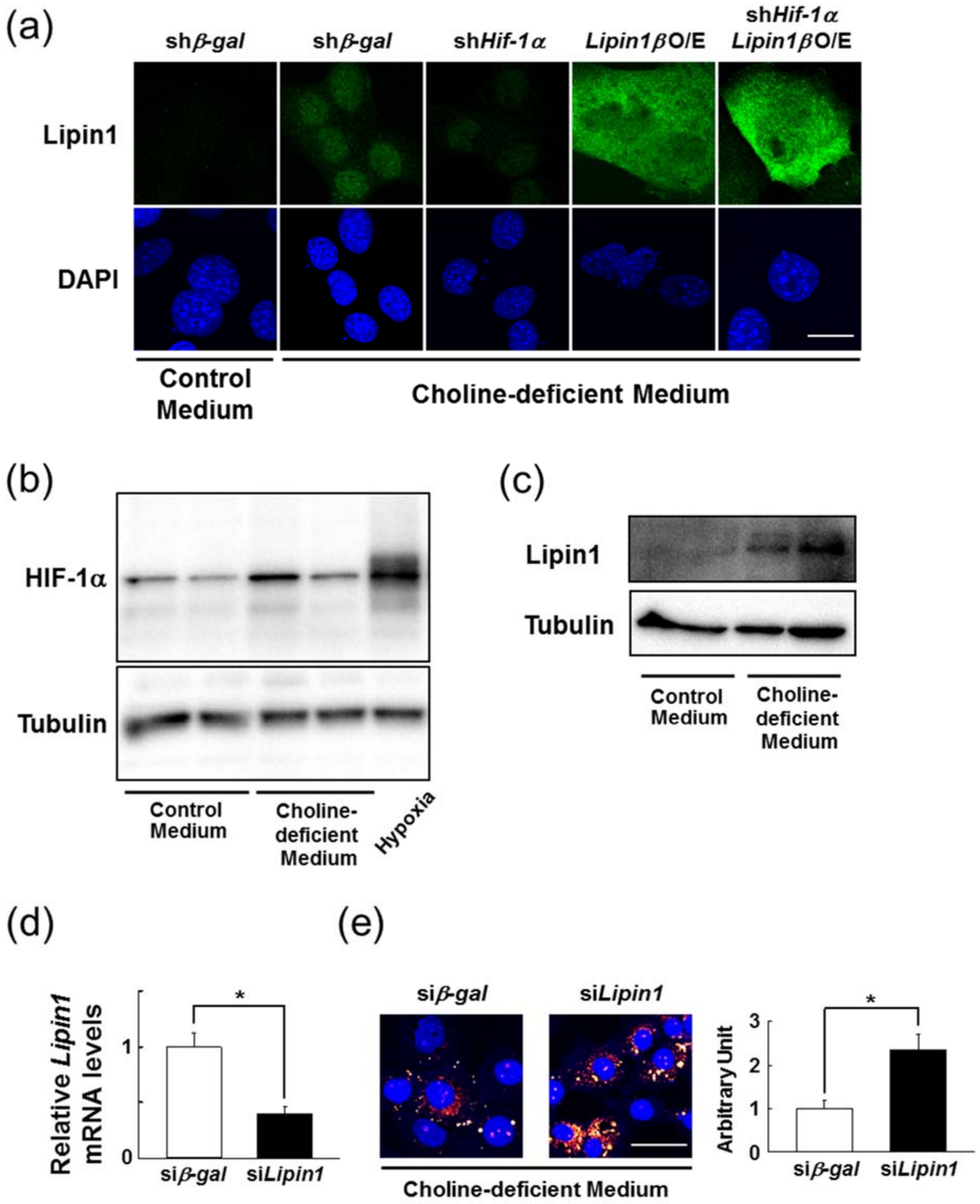

Supplementary Figure S5. Suppression of *Lipin1* exacerbates lipid accumulation caused by choline deprivation.

(a) Representative images of lipin1 (top) and DAPI (bottom) staining in AML12 cells treated with choline deficient medium. Scale bar = 20 μm.

(b), (c) Representative immunoblots of HIF-1α (b) and lipin1 (c) in AML12 cells exposed to choline deficient medium.

(d) Quantification of Lipin1 mRNA by qPCR in AML12 cells infected with adenovirus expressing shLipin1; n = 3 per group. *$P < 0.05$

(e) Representative images of Nile Red staining in Lipin1-knockdown cells treated with choline deficient medium. Red spots indicate lipid accumulation. DAPI staining is shown in blue. Scale bar is 25μm. Densitometric quantification of lipid accumulation. n=4 plates per group. *$P < 0.05$.

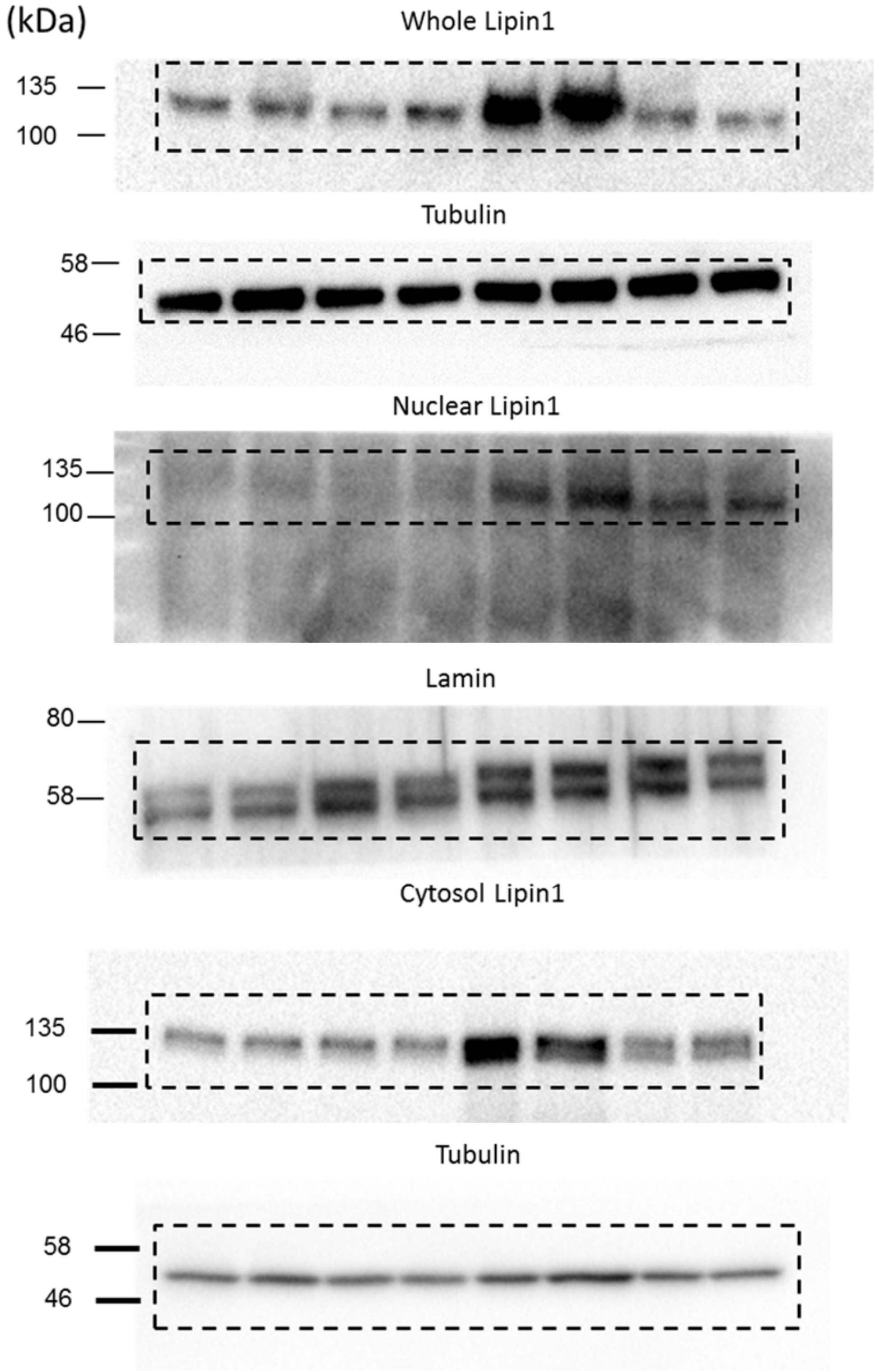

Supplementary Figure S6.

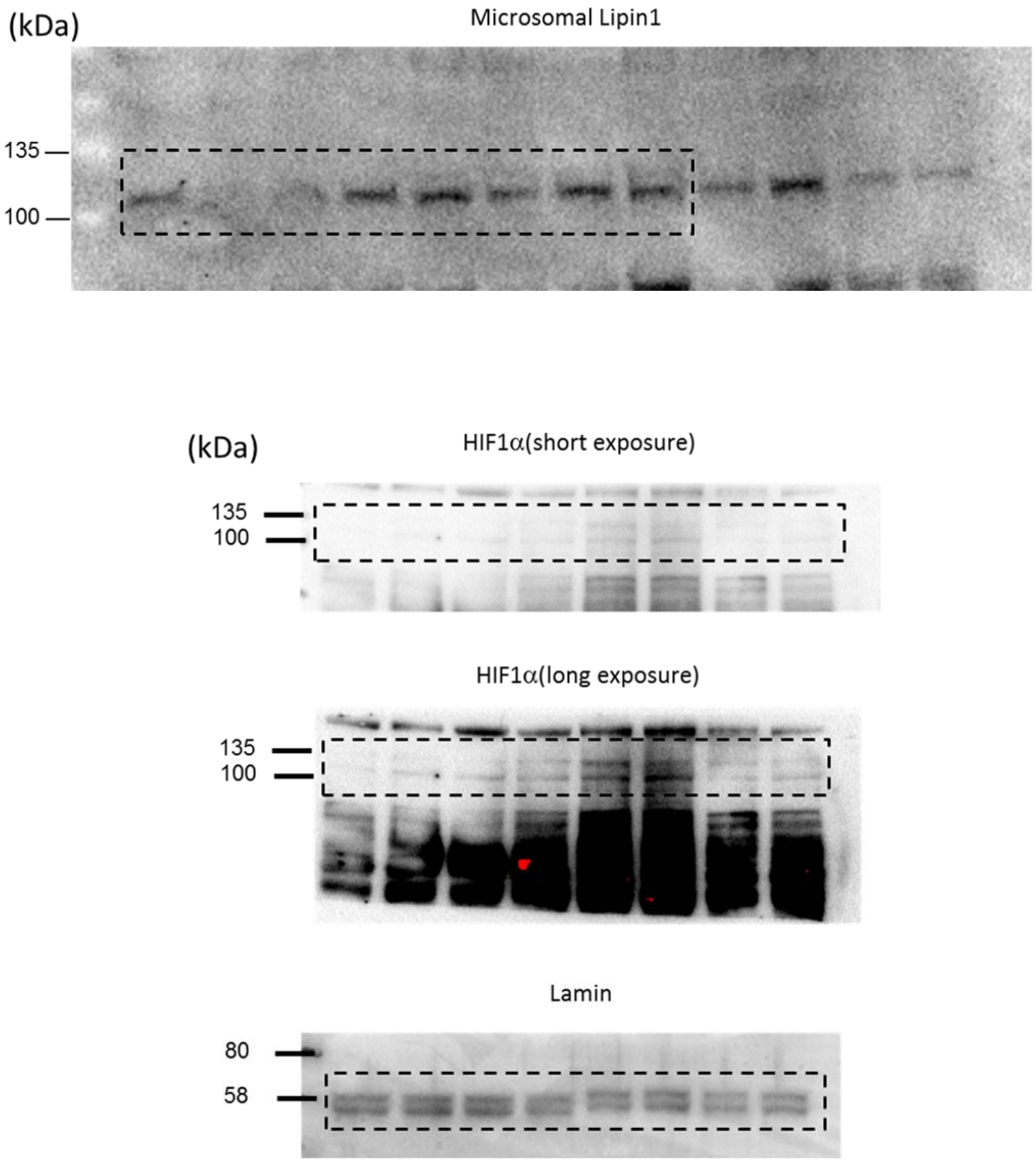

Supplementary Figure S6.

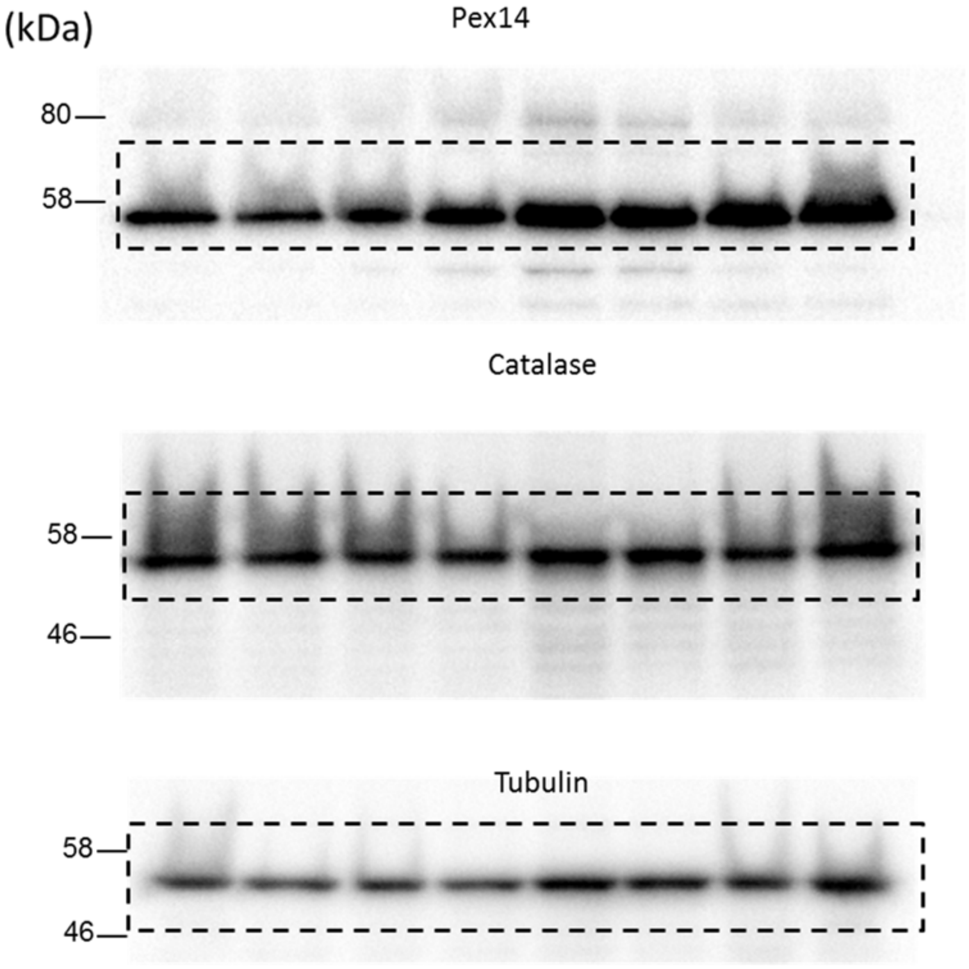

Supplementary Figure S6.

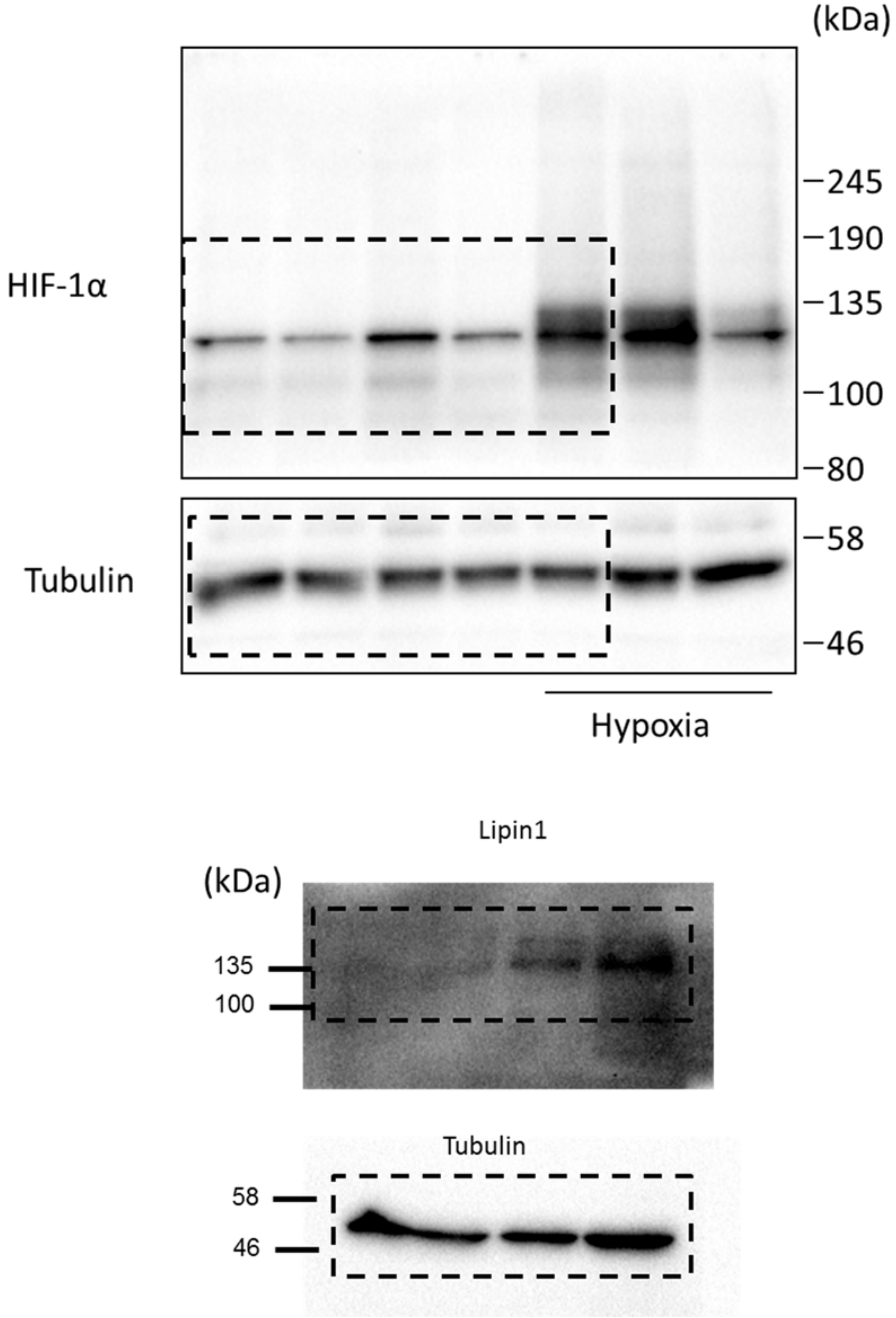

Supplementary Figure S6.

For qPCR assay

Gene name	AssayID	Gene name	AssayID	Gene name	AssayID	Gene name	AssayID	Gene name	AssayID
AOX1	Mm00443579_m1	Catalase	Mm01340251_m1	CPT1	Mm00550438_m1	LCAD	Mm00599660_m1	MCAD	Mm00431611_m1
MTP	Mm00435015_m1	PGC1α	Mm00447183_m1	PPARα	Mm00440939_m1	RXRα	Mm01332431_m1		

Gene name	Forward primer	Reverse primer	Probe
DGAT2	CTGGCTGATAGCTGTGCTCTACTT	AAAGGTGGCAGGAGATCGC	CTGGCATTTGACTGGAACACGCCC
FASN	TCCTGGAACGAGAACACGATCT	GAGACGTGTCACTCCTGGACTTG	AGCTCACGCTGCGGAAACTTCAG
SCD1	GGCCTGTACGGGATCATACTG	GGTCATGTAGTAGAAAATCCCGAAGA	TTCCCTCCTGCAAGCTCTACACCTGC
SREBP-1c	AGCCATGGATTGCACATTTG	GGCCCGGGAAGTCACTGT	AGACATGCTCCAGCTCATCAACAACCAA

Gene name	Forward primer	Reverse primer	Gene name	Forward primer	Reverse primer
Abcd1	TGCCGTGAGCATTGATGTG	TGCGATACCAGCATCTTTGG	ApoB	GAAGCGCCACCAAGATCAAC	AAGGGTACACTGGTTGGTCC
CD36	GATGTGGGCACCCTTCATG	CCGGGCTGAATTCCA	DMT1	AGGGCATGTGGCACTCTATG	CTCAGCAGGACTTTCGAGATG
FATP2	CAACACACCGCAGAAACCAA	CCATTTCCCAGGGCTTTTTT	HIF1	CACCGATTCGCCATGGA	TTCGACGTTCAGAACTCATCTTT
Lipin1	CCAAGACCAACATCAGCTCGTA	CAATGGGAAGACGTGATGCA	Lipin1α	GGTCCCCCAGCCCCAGTCCTT	GCAGCCTGTGGCAATTCA
Lipin1β	CAGCCTGGTAGATTGCCAGA	GCAGCCTGTGGCAATTCA	pex3	CCCTAGGCAACCCACACAAC	GGACTGTGCCCAGGAAGATG
pex11α	CCGAGTCGCCAACCAAAGC	GCCCAGTCTGAACCATTTAGC	pex14	AGGCCGAGAGGACAGAAAGC	GGACAGAGGCCAGTGTTGTC
PHD3	AATTGGGACGCCAAGTTACAC	GGAGGGCTGGACTTCATGTG	PPARγ	GGGATGTCTCACAATGCCATC	GGGATGTCTCACAATGCCATC
Uox	GATCAAACACGTCCATGCATTC	CGGGAGGTCCGTTTCTCATC	VEGF	AGTCCCATGAAGTGATCAAGTTCA	ATCCGCATGATCTGCATGG
β-actin	ACGGCCAGGTCATCACTATTG	CAAGAAGGAAGGCTGGAAAAGA	18S	CTTTCGCTCTGGTCCGTCTT	TTCGTATTGCGCCGCTAGA

For ChIP-qPCR assay

Gene name	Forward primer	Reverse primer
AOX-1	TCTAACGTCAGTCAAGTCGGC	CGGGGTGAGGAGGCTAATAGA
AOX-2	TGAACCTGGAGACGTGAGGT	GGCTCCTCGCCAAGGAAA
PPARα	TCAACCATCTCCAGGGTCTCAG	ACTATGCTATTTGTGGCGATGAGC
MCAD	GATTTCCTTCTCAGTCTCCT	CGGAGAAGAAGACTGTGTGC

Supplementary Table S1. Primer and probe sequences
